# Brown’s Dental Depot

**Published:** 1856-12

**Authors:** 


### BROWN'S DENTAL DEPOT.

The friends and patrons of Dr. J. M. Brown, who make but an annual winter visit to the city, will be taken with a pleasant surprise in calling on him. To be sure he is the *same* Doctor Brown, or, perhaps, *more so*, but the old stand is emphatically "more so." The Dr. has refitted and rearranged his room till it is as pretty and convenient as terrestrial stopping places should be; and as to his goods and prices, they are just such as will suit the profession, for the Doctor, after serving them so long, knows exactly what they want, and the lowest price for which it can be afforded. See first page of advertisements.

